# Structural biology and public health response to biomedical threats

**DOI:** 10.1063/4.0000186

**Published:** 2023-06-05

**Authors:** Joanna Lenkiewicz, Vanessa Bijak, Shrisha Poonuganti, Michal Szczygiel, Michal Gucwa, Krzysztof Murzyn, Wladek Minor

**Affiliations:** 1 Department of Molecular Physiology and Biological Physics, University of Virginia, Charlottesville 22908, USA; 2 Department of Computational Biophysics and Bioinformatics, Jagiellonian University, Krakow, Poland

## Abstract

Over the course of the pandemic caused by SARS-CoV-2, structural biologists have worked hand in hand with groups developing vaccines and treatments. However, relying solely on *in vitro* and clinical studies may be insufficient to guide vaccination and treatment developments, and other healthcare policies during virus mutations or peaks in infections and fatalities. Therefore, it is crucial to track statistical data related to the number of infections, deaths, and vaccinations in specific regions and present it in an easy-to-understand way.

## INTRODUCTION

At the beginning of this century, a life-threatening severe acute respiratory syndrome coronavirus (SARS-CoV) infected thousands of people. Roughly ten years later, the Middle East Respiratory Syndrome coronavirus (MERS-CoV), with approximately a 35% fatality rate, killed almost a thousand people.^1^ Scientific papers published at that time strongly suggested the possibility of a re-emergence in the future of even more deadly outbreaks of SARS-like viruses. However, the proposal for urgent studies on these viruses^2^ went almost unheeded. At the beginning of 2020, the entire world faced the threat of another coronavirus. SARS-CoV-2 was less deadly but unfortunately significantly more infectious than previous coronaviruses, quickly jeopardizing public well-being and initiating a global COVID-19 pandemic. As of February 27, 2023, over 679 × 10^6^ confirmed cases of COVID-19 and more than 6.7 × 10^6^ deaths have been reported globally. In the US alone, SARS-CoV-2 infected almost 100 × 10^6^ people and killed over 1 × 10^6^.^3^

At the beginning of 2020, many members of the scientific and medical research communities began to focus on the understanding of various properties of SARS-CoV-2. Collective efforts resulted in over 300 000 scientific publications and over 2700 structures of SARS-CoV-2 and its fragments deposited in the PDB.^4^ Scientists quickly sequenced and annotated the virus's genome and made unprecedented progress in understanding its mechanism of action. Finally, three decades of work by hundreds of scientists on messenger RNA vaccines^5^ served as the basis for some novel vaccines against the virus. In December 2020, the administration of vaccines for SARS-CoV-2 began.^6^ The FDA initially authorized three COVID-19 vaccines for public use in the US: mRNA vaccines developed by Pfizer-BioNTech and Moderna,^7^ and an adenovirus-based vaccine developed by Johnson and Johnson.^8^ In July 2022, the FDA approved a fourth, described as a protein subunit and virus-like particle, vaccine by Novavax.^9^

Clinical trials followed by large-scale initial vaccinations have proved the efficacy of the vaccines against COVID-19 infection;^10,11^ however, later studies showed^12^ that they are less effective against some newly emerging variants. Unfortunately, a significant fraction of the US population was never convinced that vaccinations and masking were necessary from the pandemic's start. Misinformation was amplified through social media, generated by poor quality and often contradictory data from decentralized and underfunded database systems. During the onset of the COVID-19 pandemic, various studies revealed that between 15% and 37% (depending on the platform studied) of social media users believed that stories classified as misinformation were true.^13,14^ It is important to stress that not only does the prevalence of public misinformation matter, but also the influence of the people who spread it. More than three years after the start of the COVID-19 pandemic, many scientists, companies, and government officials are still struggling to understand why only about 14% of US residents have received the bivalent vaccine.^15^

Many resources were created and made publicly available to track the daily number of new infections and deaths caused by COVID-19. These sources are available locally, nationally, and globally. An example of a local reference is the UVA COVID tracker,^16^ a tool created to track the progress of COVID-19 within the University of Virginia community. Two crucial national sources in the US are the Centers for Disease Control and Prevention^17^ and USAFacts.^18^ Two important global tools for tracking COVID-19 data are the JHU COVID Tracker^19^ and Worldometer.^20^ The JHU COVID Tracker offers real-time information on the number of confirmed cases, deaths, and recoveries worldwide as well as detailed statistics for individual countries and regions. Its advanced data visualization tools and detailed statistics make it a valuable resource for in-depth analysis. On the other hand, Worldometer provides an overview of global COVID-19 numbers with a simpler user interface that is easy to understand for a broader audience.

The data that feed these organizations and databases come from various healthcare facilities. However, these local database systems are often plagued by numerous design flaws and inefficiencies. Entering data into databases is seen as a bureaucratic nuisance and not a critical task necessary to make decisions based on transparent and verifiable data. Datasets come from many different locations, in different formats (paper and electronic) and at different time intervals. This makes the data collection process prone to human error and often results in outdated data on infections, hospitalizations, deaths, and immunizations. As one epidemiologist stated in the *Washington Post*: “[]we are flying blind.”^21^ In the *Washington Post* article, the authors describe the system for collecting and organizing data regarding the COVID-19 pandemic as “a largely 19th-century system” (which obviously is an exaggeration). As a result, the public is underinformed about how effective the first two boosters were and why it is necessary to take a bivalent booster now. The intelligent data mining of aggregated data is a daunting task that requires collecting data from multiple, sometimes disparate, sources. In this paper, the authors evaluate data on the efficacy of COVID-19 vaccines. We present examples of problems with COVID-19 pandemic data, along with the types of errors that caused these problems. Finally, this manuscript proposes relatively simple solutions for data cleaning and analysis. We also offer a long-term solution: creating an advanced information system (AIS) that would be adequate to fight future pandemics.

## MATERIALS AND METHODS

The data about deposits of structures, publications, and other information related to structural biology were mainly obtained from public wwwPDB databases.^22,23^ The Web of Science API^24^ and PubMed^25^ were used as a source of information about publications and citations. County-level data about cases, deaths, and population are acquired from USAFacts.^18^ County-level vaccination data are sourced from the Centers for Disease Control and Prevention (CDC).^15^ Land area data for US counties were obtained from OpenIntro.^26^ Educational data were obtained from Census.^27^

Data analysis and visualization were performed in Python,^28^ using the pandas,^29^ NumPy,^30^ matplotlib,^31^ seaborn,^32^ and selenium^33^ libraries. For easy regeneration of various reports and visualizations, the analysis was done using Jupyter notebooks.^34^

## RESULTS

### COVID-19 and structural biology

Structural biologists were among the first to respond to the emerging threat caused by SARS-CoV-2, even before the general scientific community learned about the virus. When scientists characterized the genome of the virus by next-generation sequencing on samples obtained from infected patients,^35^ Zihe Rao from Tsinghua University in Beijing expressed SARS-CoV-2 main protease, and his group purified and crystallized a diffraction-quality sample within a few days, with structure determination and refinement following shortly thereafter. The first version of the PDB deposit was released on February 5, 2020, with the PDB access code 6LU7, weeks before COVID-19 became a global pandemic. The deposit was not perfect—the Rao group subsequently submitted 12 more revisions of the same structure—but even the first deposit allowed others to significantly speed up the use of structural information for follow-up studies. The paper describing this structure was published in *Nature* three months later.^36^

As of the end of February 2023, over 2700 SARS-CoV-2 related structures have been deposited in the PDB. This is impressive, especially compared to the structural work on other life-threatening diseases (see Table I). After 25 years of study, HIV-related structures have roughly only 2900 structural models in the PDB. Other infectious diseases have a much smaller number of structural models in the PDB. The pace of deposition has been so high that many valuable structural models were deposited long before peer-reviewed publication.

**TABLE I.** The number of deposits, primary publications, citations, and publications in PubMed of most common diseases as of February 2023.

Targets	Year of first primary citation	Deposits	Primary publications	Citations	Publications in PubMed	Primary publications/PubMed publication (%)	Primary publications/deposits (%)
SARS-CoV-2	2020	2770	481	49 463	256 887	0.19	17.36
Zika	2016	161	60	8116	7066	0.85	37.27
Ebola	1998	131	60	4165	6780	0.88	45.80
Herpes	1997	129	63	3817	35 990	0.18	48.84
Malaria	1996	113	50	5461	52 202	0.10	44.25
Tuberculosis	1995	2620	810	26 551	155 170	0.52	30.92
Influenza	1990	1415	443	36 735	77 988	0.57	31.31
HIV/AIDS	1989	2926	1028	86 405	265 827	0.39	35.13

**FIG. 1.**
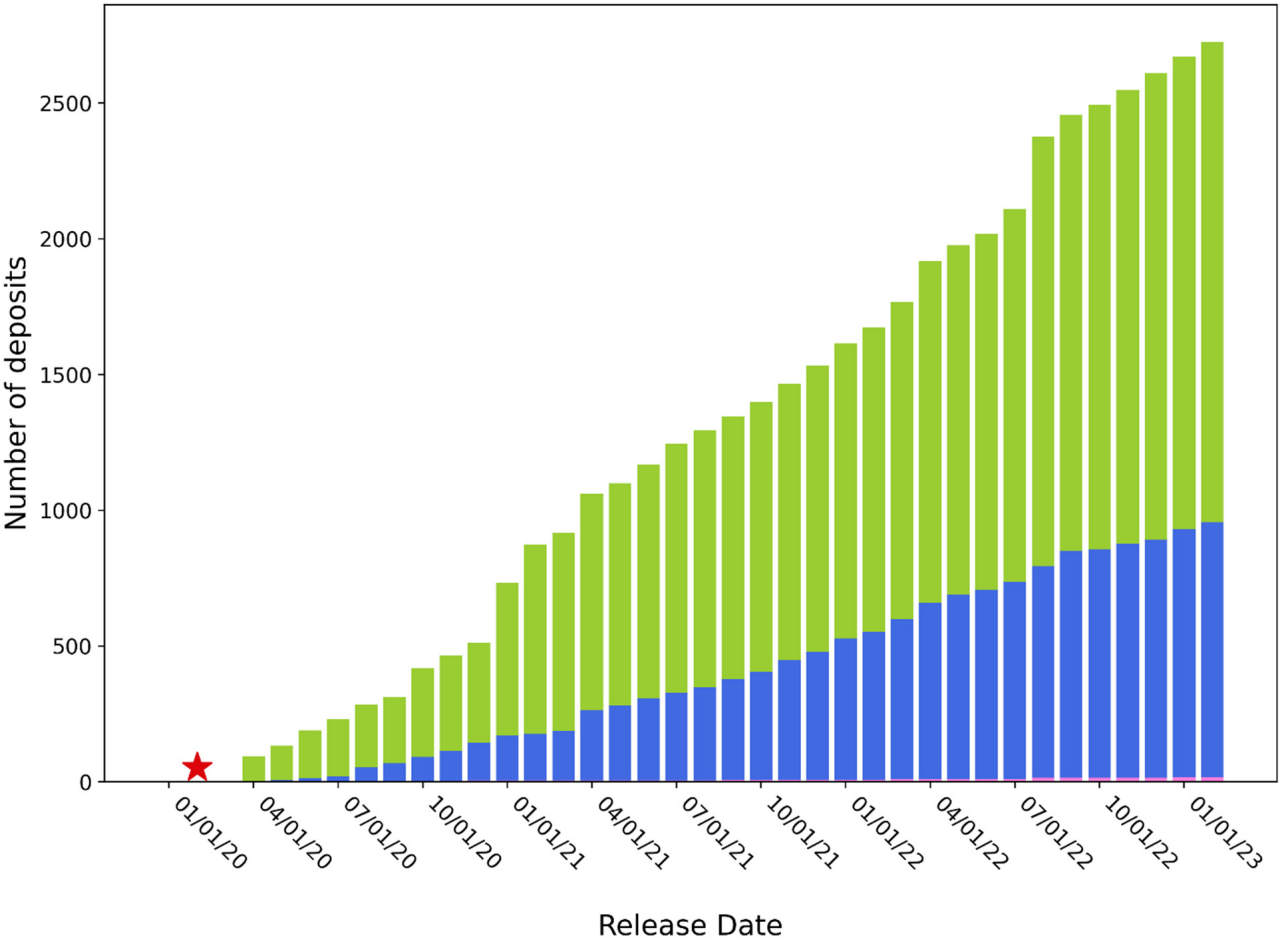
The number of structural models of various components of SARS-CoV-2 determined using x-ray crystallography (green), cryo-EM (blue), and other methods (pink). The red star corresponds to the first structure PDB ID 6LU7.

**FIG. 2.**
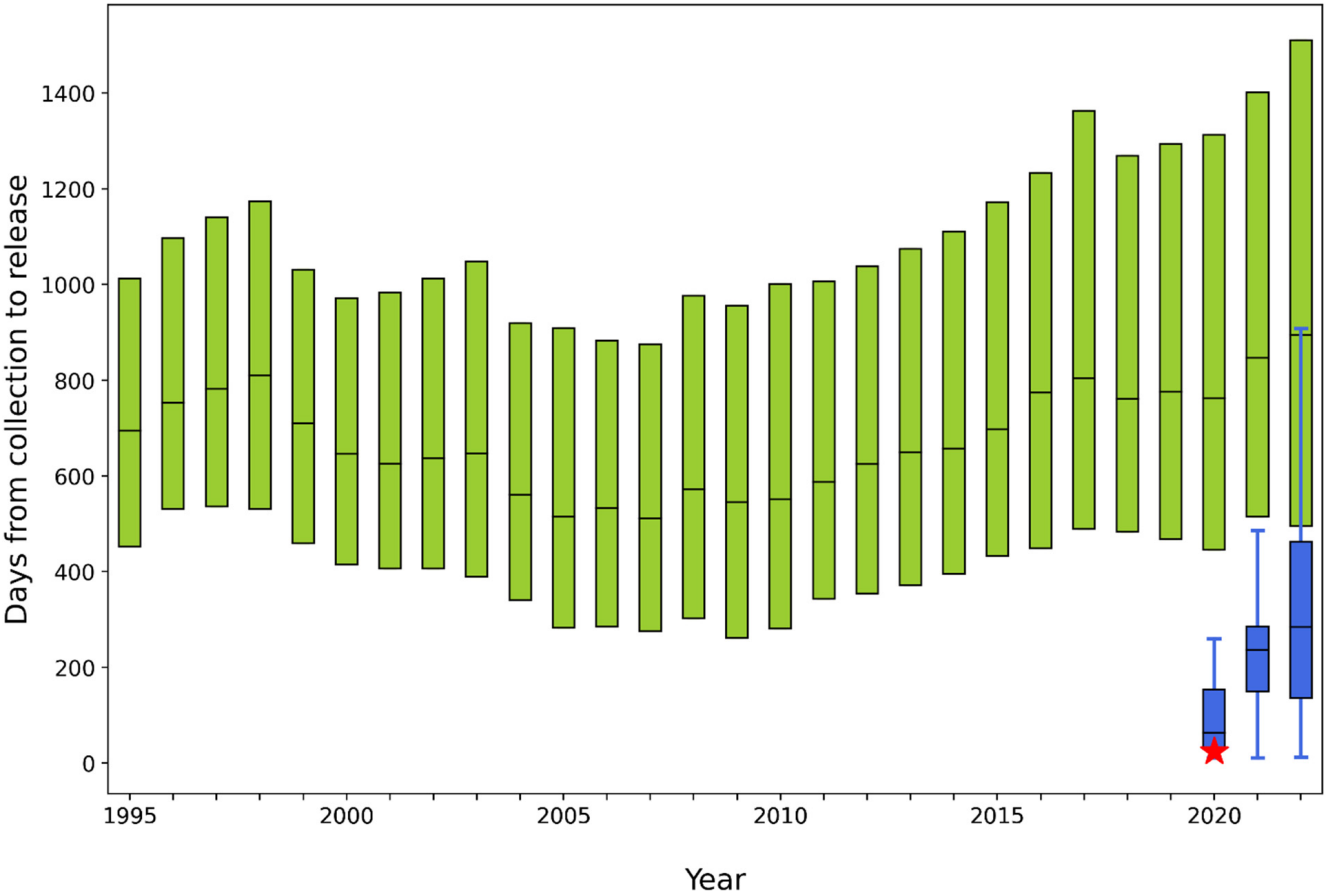
Number of days between data collection and release of PDB deposits for all structures (green boxes cover 50% of data) and SARS-CoV-2 structures (blue boxes cover 50% of data and whiskers represent 80% of data). The red star corresponds to PDB ID 67U7.

**FIG. 3.**
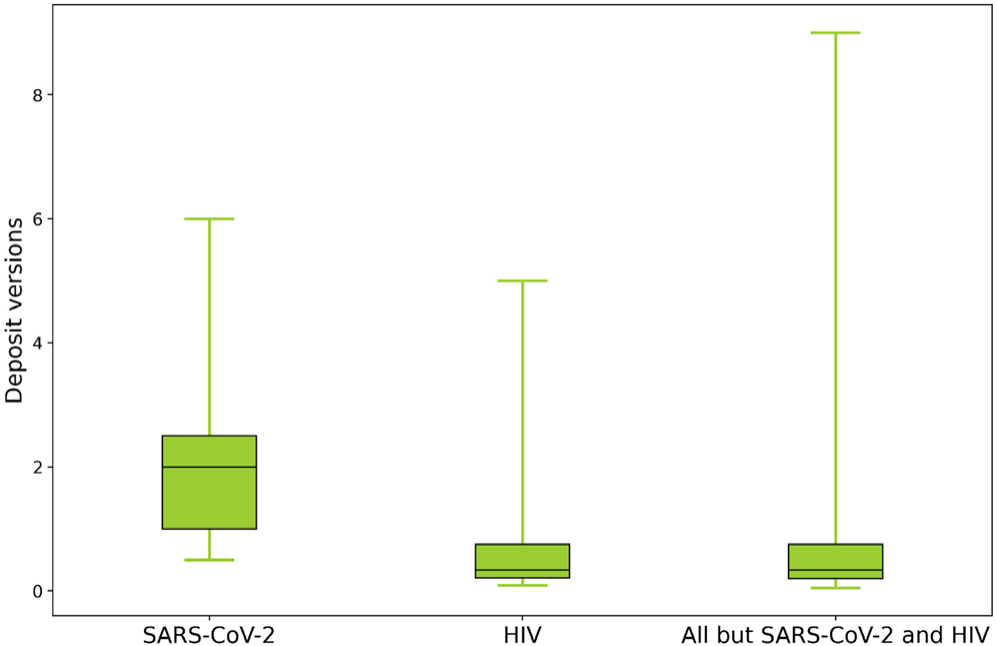
The number of PDB deposit versions per year for SARS-CoV-2, HIV, and all deposits excluding SARS-CoV-2 and HIV. Boxes represent 50% of data, and whiskers mark the range between minimum and maximum number of versions per year.

After the first structure was deposited in February 2020, the number of structural models of various components of SARS-CoV-2 determined using mainly x-ray and cryo-electron microscopy (cryo-EM) increased very quickly (Fig. 1). Simultaneously, researchers accelerated the dissemination of their research. In structural biology, this meant shortening the time between data collection and deposition of the structural model to the PDB. Most of the COVID-19-related structures were deposited without a time embargo and before publication. Examination of the time between data collection and release shows that this time was significantly shortened compared to non-SARS-CoV-2 structures (Fig. 2). In 2021, this time was two times shorter than the average time in 2005 (when the Protein Structure Initiative (PSI) centers were evaluated). Such an increase in speed increases the risk of error, which could slow down rather than speed up drug development. Several groups carefully reexamined COVID-19 related structural models.^37,38^ Some of these groups attempted to organize them in a way that is easily accessible, simple to understand, and valuable not only to structural biologists but also to a broader scientific community. It was shown that faster structure determination did not negatively affect the overall structure quality. According to the P_Q1_ metric,^39^ the overall quality of SARS-CoV-2 structures is similar to other structures determined during the same time frame. The COVID-19.bioreproduciblity.org website^38,40,41^ focused on assessing the small-molecule ligands modeled in COVID-19 structural models, as such information provides invaluable hints for inhibitor search. The extensive use of state-of-the-art validation tools^42^ resulted in optimization of several re-deposited models in collaboration with the original authors. The tools and re-refinement protocols used in this project can serve as a template for future structure assessment efforts. Careful examination of COVID-19 structural results urged many authors of SARS-CoV-2-related deposits to examine their structural models and sometimes submit several versions of their deposits. Roughly 11% of SARS-CoV-2 related depositors of entries have submitted experimental diffraction images to the Integrated Resource for Reproducibility in Macromolecular Crystallography.^43,44^ Sometimes even a request for access to diffraction data motivated researchers to examine deposits followed by the deposition of a new version. The average number of versions (per year) for SARS-CoV-2 deposits is three times higher than for other deposits (Fig. 3).

The number of citations of primary publications reflects the importance of structural data for subsequent academic research. Table II shows the ten most cited papers. The top paper^45^ is cited almost as many times as the paper describing research^46^ that led to a Nobel Prize. One can say that during the pandemic, the number of citations is much less significant than using scientific information to develop treatments to combat the public health threats; however, papers from Table II had information critical for medical developments. Structures described in those publications provide critical information about the virus's mechanism of action and potential drug targets. The structure of the spike protein of SARS-CoV-2^45^ (PDB ID 6VXX) has played a crucial role in the development of COVID-19 vaccines.^47^ The Pfizer-BioNTech and Moderna mRNA vaccines both utilize the spike protein structure to trigger an immune response against the virus. The main protease structure of SARS-CoV-2 (PDB ID 6VYB) has been used to design drugs that specifically target this enzyme and prevent virus replication. One such drug is molnupiravir,^48^ which was approved by the FDA for the treatment of certain COVID-19 cases. The RNA-dependent RNA polymerase (RdRp) of SARS-CoV-2 (PDB ID 6M0J) has also been used to design drugs that target this enzyme and inhibit virus replication. The FDA-approved drug, remdesivir,^49^ is one such example of a drug that was developed with the help of this structure.

**TABLE II.** Ten most cited COVID-19 publications in structural biology as of February 15, 2023.

Title	Authors	PMID	PDB ID	Date	Number of citations
Structure, function, and antigenicity of the SARS-CoV-2 spike glycoprotein.	Walls *et al.*	32 155 444	6VXX 6VYB	April 2020	4891
Structure of the SARS-CoV-2 spike receptor-binding domain bound to the ACE2 receptor.	Lan *et al.*	32 225 176	6M0J	March 2020	3009
Structural basis for the recognition of SARS-CoV-2 by full-length human ACE2.	Yan *et al.*	32 132 184	6M17 6M18 6M1D	March 2020	2795
Structural basis of receptor recognition by SARS-CoV-2.	Shang *et al.*	32 225 175	6VW1	March 2020	1998
Structure of M^pro^ from SARS-CoV-2 and discovery of its inhibitors.	Jin *et al.*	32 272 481	6LU7 7BQY	April 2020	1959
Crystal structure of SARS-CoV-2 main protease provides a basis for design of improved α-ketoamide inhibitors.	Zhang *et al.*	32 198 291	6Y2G 6Y2F 6Y2E 6Y7M	March 2020	1686
Structural and Functional Basis of SARS-CoV-2 Entry by Using Human ACE2.	Wang *et al.*	32 275 855	6LZG	May 2020	1645
Human neutralizing antibodies elicited by SARS-CoV-2 infection.	Ju *et al.*	32 454 513	7BWJ	May 2020	890
Cross-neutralization of SARS-CoV-2 by a human monoclonal SARS-CoV antibody.	Pinto *et al.*	32 422 645	6WPS 6WS6 6WPT	May 2020	880
A highly conserved cryptic epitope in the receptor binding domains of SARS-CoV-2 and SARS-CoV.	Yuan *et al.*	32 245 784	6W41	April 2020	811

From a scientific point of view, the most impressive development was the creation of the sotrovimab^50^ treatment based on a natural antibody discovered in the blood of a COVID-19 patient. Sotrovimab is a therapy developed by a large international team of 50 researchers from 14 institutions. Combining x-ray crystallography and cryo-EM studies elucidated ways of how these antibodies bind to the SARS-CoV-2 spike protein. An antibody called S309 neutralized not only all known SARS-CoV-2 strains but also the original SARS-CoV. Due to very promising *in vitro* and clinical studies, sotrovimab received emergency use authorization from the FDA in May 2021. However, in the field, the therapy did not demonstrate the same level of efficacy with new strains. As of April 5, 2022, Sotrovimab is no longer authorized by FDA to treat COVID-19 in any U.S. region due to an increase in cases caused by Omicron BA.2 subvariant.^51^ It seems that coronavirus vaccines and treatments must be closely matched to circulating strains to provide reasonable protection. This case illustrates that even the most ideal *in vitro* and clinical studies do not necessarily address the complex interplay between a virus and a patient's immune system. This phenomenon thoroughly displays the importance of in-depth observation of treatments and vaccine use and careful, extensive analysis of all available data. However, current national databases contain inconsistencies, design flaws, and outright bugs^52^ that do not allow for detailed analysis of treatments and vaccinations. Structural biologists often complain about various structural biology data mishaps, but our experience shows that structural biology is an absolute leader in data quality, management, and transparency. For the well-being of humanity, we should leave the structural biology ivory tower and critically investigate the data that do not belong to structural biology but are closely related to our research.

### Evolution of SARS-CoV-2 and its influence on the development of vaccines and vaccinations across the US

**FIG. 4.**
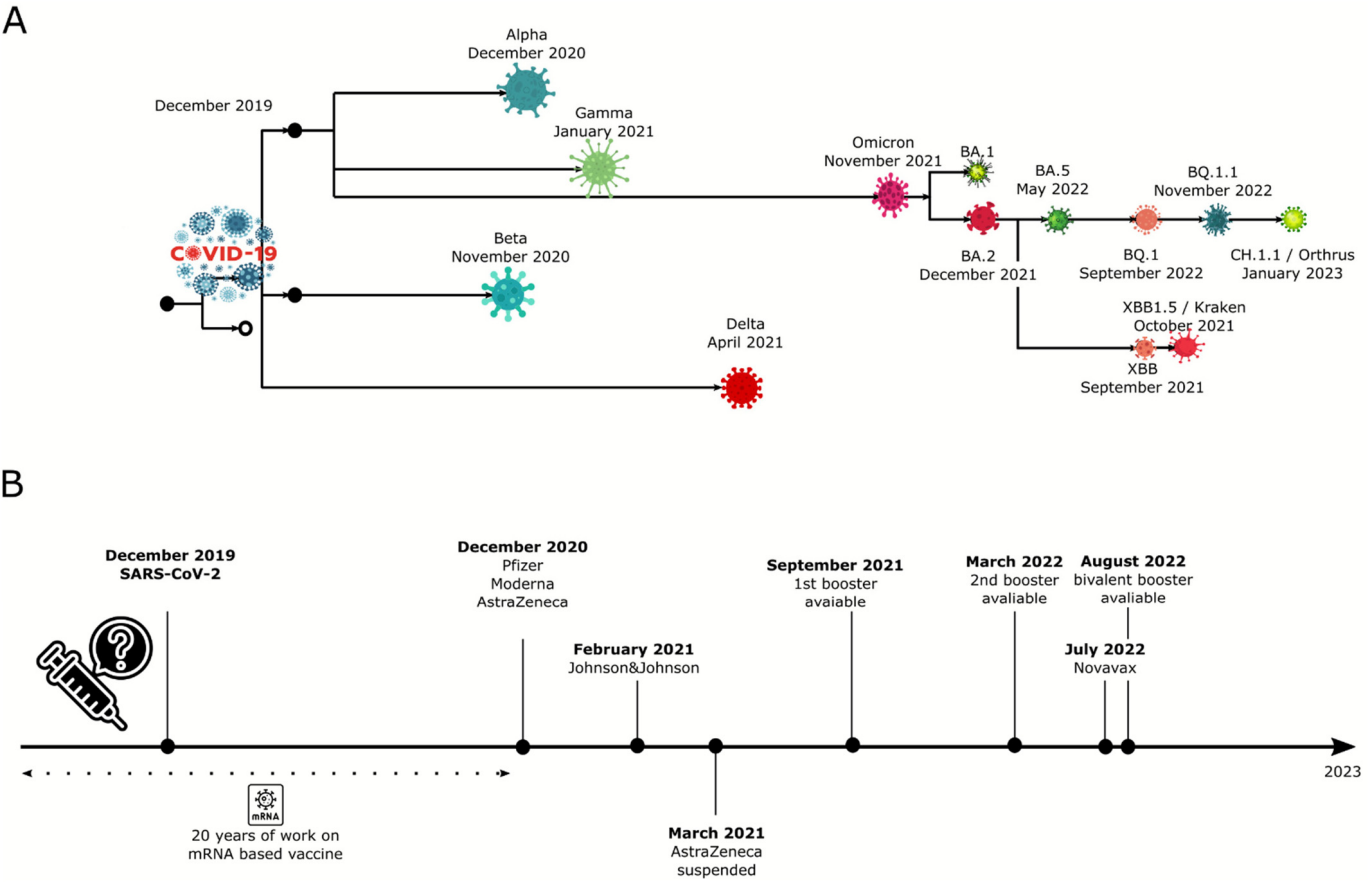
Timeline of the development of COVID-19 vaccines and growing family of new COVID-19 strains for five variants of concern.

A key contributing factor to the COVID-19 pandemic's lasting effect on the world is the emergence of different strains and variants over the past three years. A timeline of five variants that were recognized during the pandemic is presented in Fig. 4. Additionally, the figure shows the sub lineages of Omicron variants such as BA.2, BA.5, BQ.1, and BQ.1.1. The timeline highlights the short intervals between identifying new variants. The beta lineage was recognized and identified in November 2020, and the alpha lineage following in December 2020. This has many implications: one being the possible infection or reinfection of those who have already been vaccinated or infected with COVID-19. This phenomenon provides the rationale for creating booster vaccines. The bottom half of Fig. 4 presents the timeline for developing COVID-19 vaccines. These two timelines may be compared to show that the booster vaccines were released soon after new variants were discovered and shown to be a serious threat. For example, the latest bivalent booster that was released for public usage in August 2022 was created in response to the BA.4/BA.5 Omicron subvariants that emerged in early 2022. However, the BQ.1.1 strain arose later in November 2022. Unfortunately, with the bivalent vaccine being released before the arrival of BQ.1.1, additional data ought to be collected to evaluate the bivalent booster's effectiveness on that strain and any subsequent strains that may arise.

**FIG. 5.**
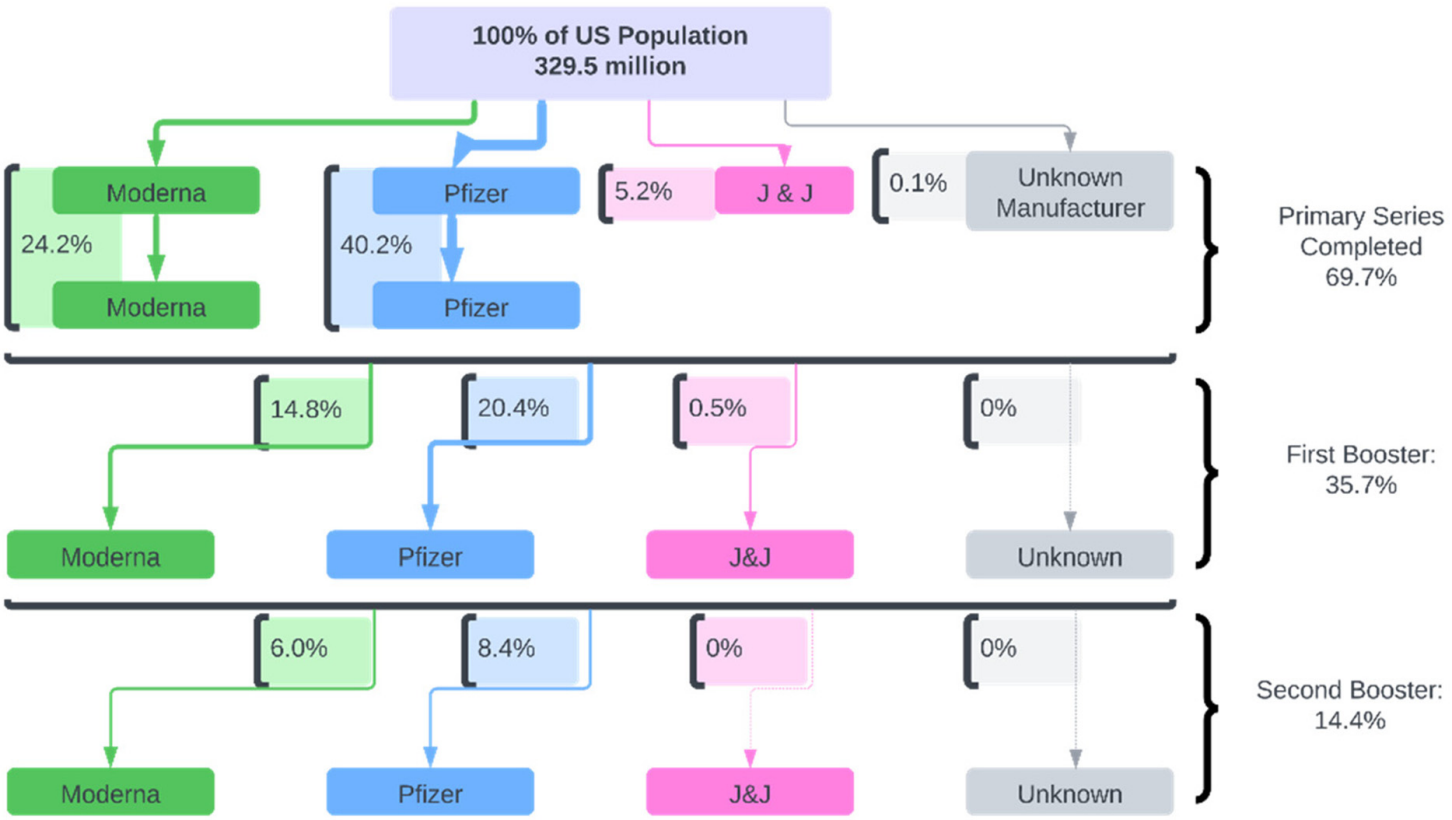
Diagram with quantified data regarding vaccination in the USA. (Data obtained from CDC on February 15, 2022.)

Figure 5 shows the percentage of vaccinated and unvaccinated people within the US and depicts the quantification of vaccine pathways. The fact that about 30% of US citizens are unvaccinated could potentially be associated with the issue of vaccine hesitancy. There could also be other reasons, such as the age restrictions preventing children from receiving the vaccines at the start of the pandemic. On the selection of vaccines, Pfizer and Moderna had similar usage rates, while Johnson and Johnson was less likely to be used. The type of vaccine is also necessary to note as Pfizer/BioNTech and Moderna vaccines are both mRNA-based vaccines, and Johnson and Johnson is adenovirus-based. This distinction, in addition to the timeline of when the vaccines were released, may have contributed to the difference in the percentage of administration at the various levels observed above. Moving to the booster shots, we can see a significant drop in vaccine compliance. The first booster's overall percentage was 35.7%, dropping drastically to 14.4% for the second booster shot. This can be due to numerous reasons, such as personal choice in taking the booster vaccine and possible misguidedness of who was meant to take the booster. For instance, there was little information provided to the public from the CDC regarding whether those who received the J&J primary series should get the Pfizer or Moderna booster. Furthermore, there was little to no information regarding the blending of vaccines and whether it is beneficial during the pandemic or safe for individual health.

### Vaccination hesitancy and impact of COVID-19 vaccination on the number of deaths and infections

**FIG. 6.**
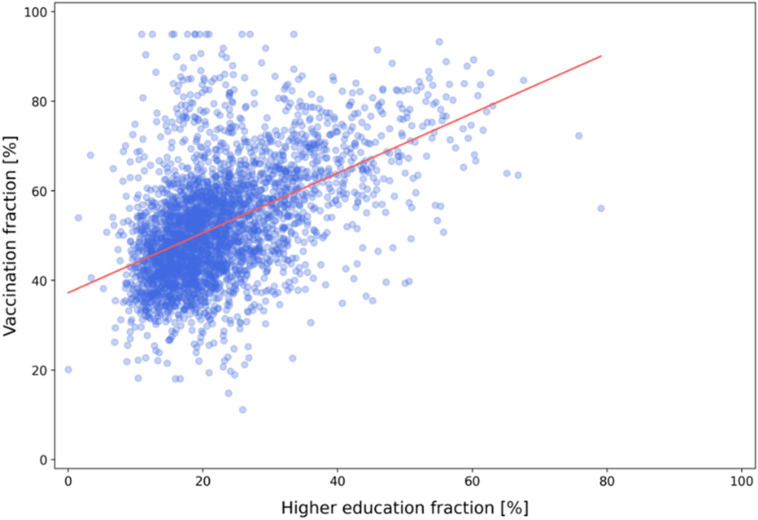
Relation between higher education fraction (percentage of people with at least a bachelor's degree) and vaccination fraction by US county. The orange line represents the linear fit to experimental data. Data were obtained in June 2022.

Vaccine hesitancy, a delay in acceptance or refusal of vaccines despite the availability of vaccination services, has existed since the first vaccines were developed. People who are not up to date with scientific developments doubt about novel solutions and the quick pace of current scientific advancements. This creates fertile ground for conspiracy theories that people easily pick up. We analyzed the relationship between the fraction of the population with higher education and the fraction of vaccinated people in several US counties. As presented in Fig. 6, there is a positive correlation (Spearman and Pearson correlation coefficients were 0.485 and 0.498, respectively) between vaccination and higher education fractions. Higher education is defined as people with a bachelor's degree and above. This shows that vaccine hesitancy may often be result of a lack of knowledge about vaccines mechanism of action. Some people are more likely to follow mass media or social networking sites, with easy-to-understand articles or videos, instead of dry scientific facts, which are more difficult to comprehend.

**FIG. 7.**
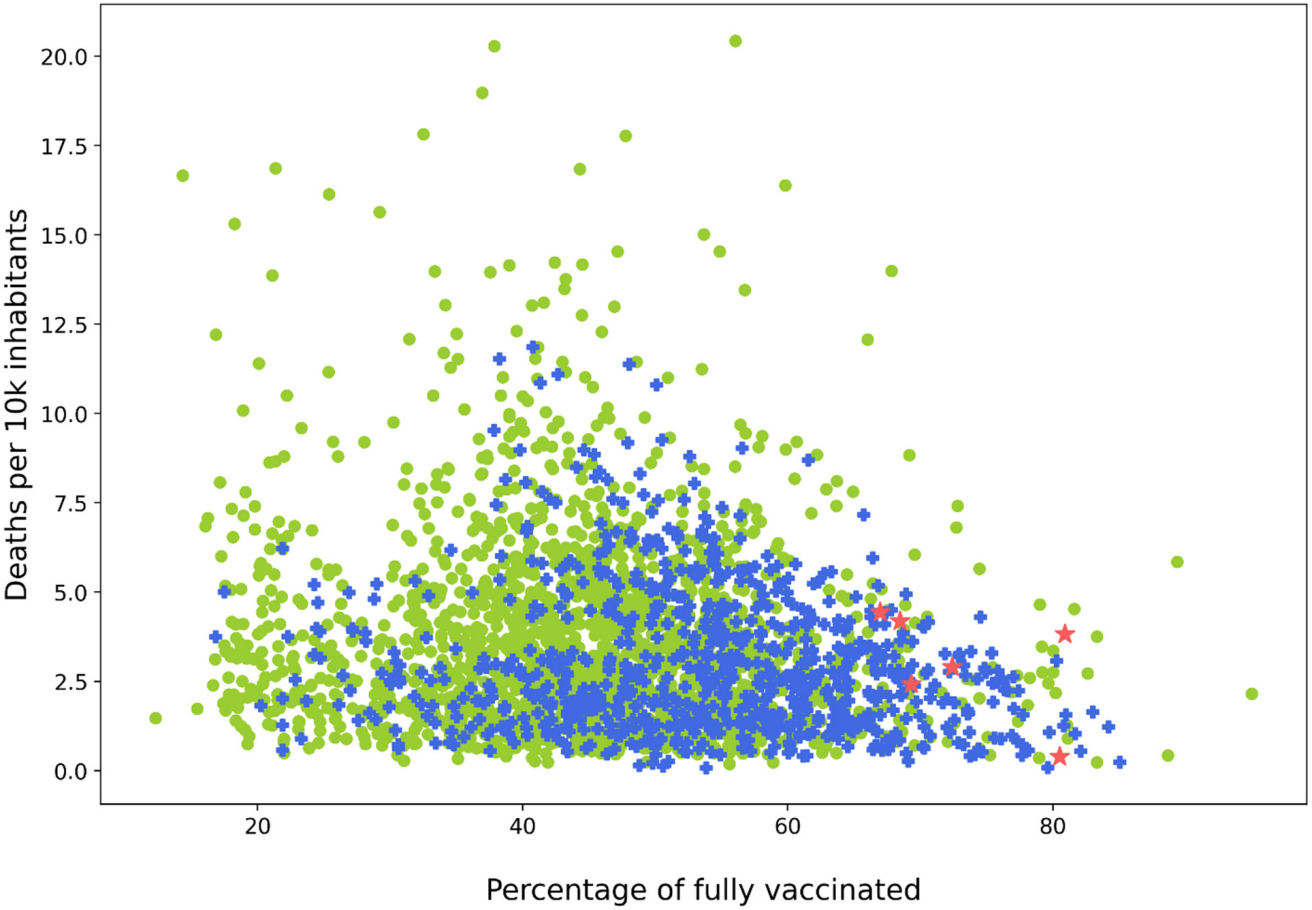
The number of COVID-19 deaths per 10 000 inhabitants vs the percentage of fully vaccinated inhabitants in US counties. The markers represent the population's density: green dots—below 100, blue crosses—above 100 and below 12 000, and red stars—above 12 000. The data represented the period between May 1, 2021 and January 31, 2022 and were downloaded in June 2022 from CDC and USAFacts.

The last available vaccination, infections, and mortality data for most counties were reported in June 2022. At that time, in some regions, the vaccination rate was dangerously low. We examined the correlation between the vaccination rate and the COVID-19 mortality rate (Fig. 7), where we showed the relationship between deaths caused by COVID-19 per 10 000 inhabitants vs the percentage of fully vaccinated population in every US county. The message is clear: lower COVID-19 mortality is dependent on an increased vaccination rate.

**FIG. 8.**
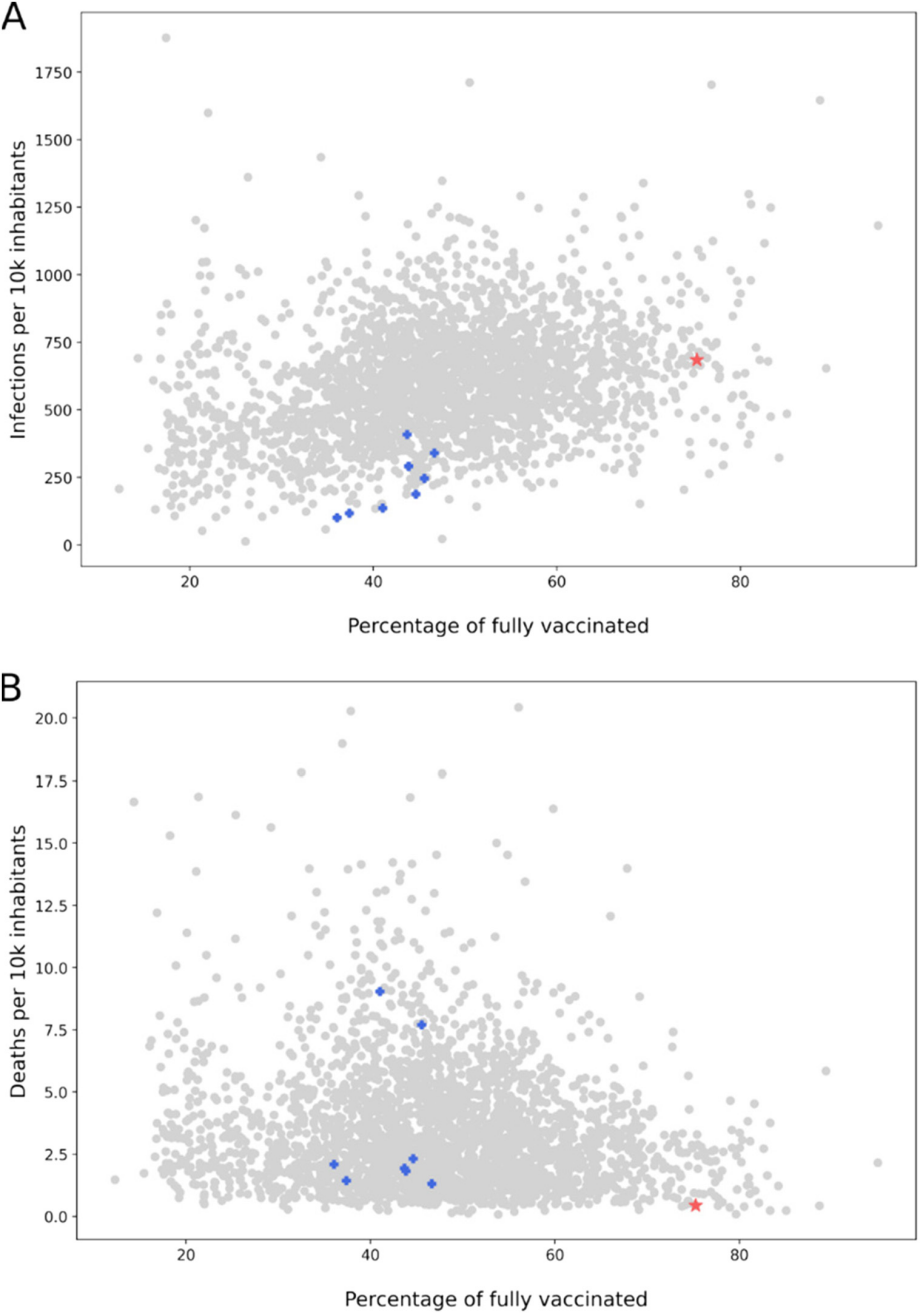
The number of infections (A) and deaths (B) caused by COVID-19 per 100 000 inhabitants versus the percentage of fully vaccinated inhabitants in US counties. Blaine county is marked using red star, and its neighbors are marked with blue crosses. The data present the period between May 1st, 2021, and January 31st, 2022, and was downloaded in June 2022 from CDC and USA Facts.

The prevalence of COVID-19 in one county can also be influenced by the situation in its neighboring counties. Frequently, people work in a different county than the one in which they live, and they can interact with the infected people in places outside their home county. This phenomenon is accurately demonstrated by the example of Blaine County, Idaho. Sun Valley, a famous ski resort, is in the Blaine County, but most of the resort staff live in neighboring counties. Blaine County had many infections despite having a high rate of vaccination. Yet, its neighboring counties, where most of its employees reside, have a low vaccination rate. As for the death rate, it is low in Blaine County but high in its neighboring counties. The situation is shown in Fig. 8.

### Problems with the collection and storage of the pandemic data

While deliberate misinformation has been a concern throughout the pandemic, plentiful misinformation has also been generated by poor quality and often contradictory data from decentralized, underfunded database systems. Design errors and inefficiencies often plague local database systems. Errors found in major repositories like the CDC or USAFacts can raise doubts about the integrity of researchers, government bodies, etc. This section explores and displays the problems we encountered while analyzing the data acquired from major COVID-19 data sources: CDC and USAFacts.

**FIG. 9.**
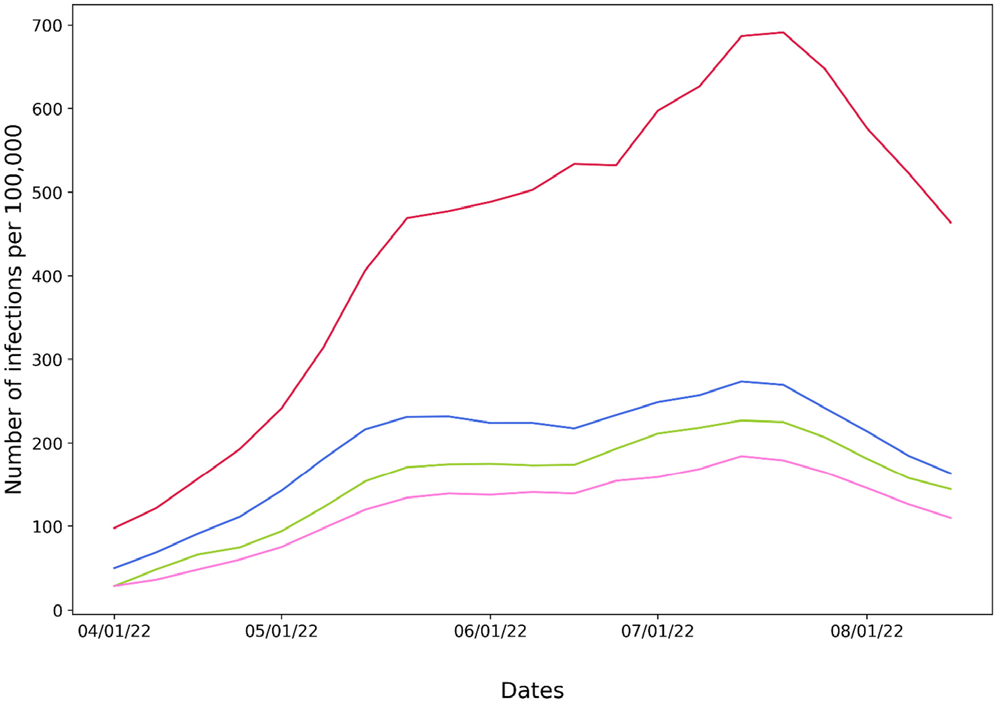
Number of infections per 100 000 inhabitants for people vaccinated with primary series (pink), primary series with one booster shot (blue), primary series with two booster shots (green), and unvaccinated people (red). Data were obtained from CDC in October 2022.

One of the prime questions regarding COVID-19 strategies is the efficacy of booster shots. Data analysis could help improve our understanding of who should receive booster shots and when and how often this should occur. We might assume that receiving two booster shots would provide the best protection also against virus infection. However, we found data that contradict this assumption. Figure 9 shows that the number of infections per 100 000 inhabitants for unvaccinated is significantly higher than for vaccinated, which is an expected outcome. However, when comparing the infections per 100 000 inhabitants among those vaccinated, COVID-19 cases per 100 000 inhabitants are higher among people who were vaccinated with either one booster or two boosters compared to those vaccinated with only the primary series. This does not necessarily suggest that the booster vaccination is not effective but rather reflects faults in the database design. The boosters were provided to older and immunocompromised people, and these groups were not separated from the entire population. Moreover, vaccine shots are effective after a period of time (generally more than two weeks), and we are not able to identify the time between infection and vaccination.

**FIG. 10.**
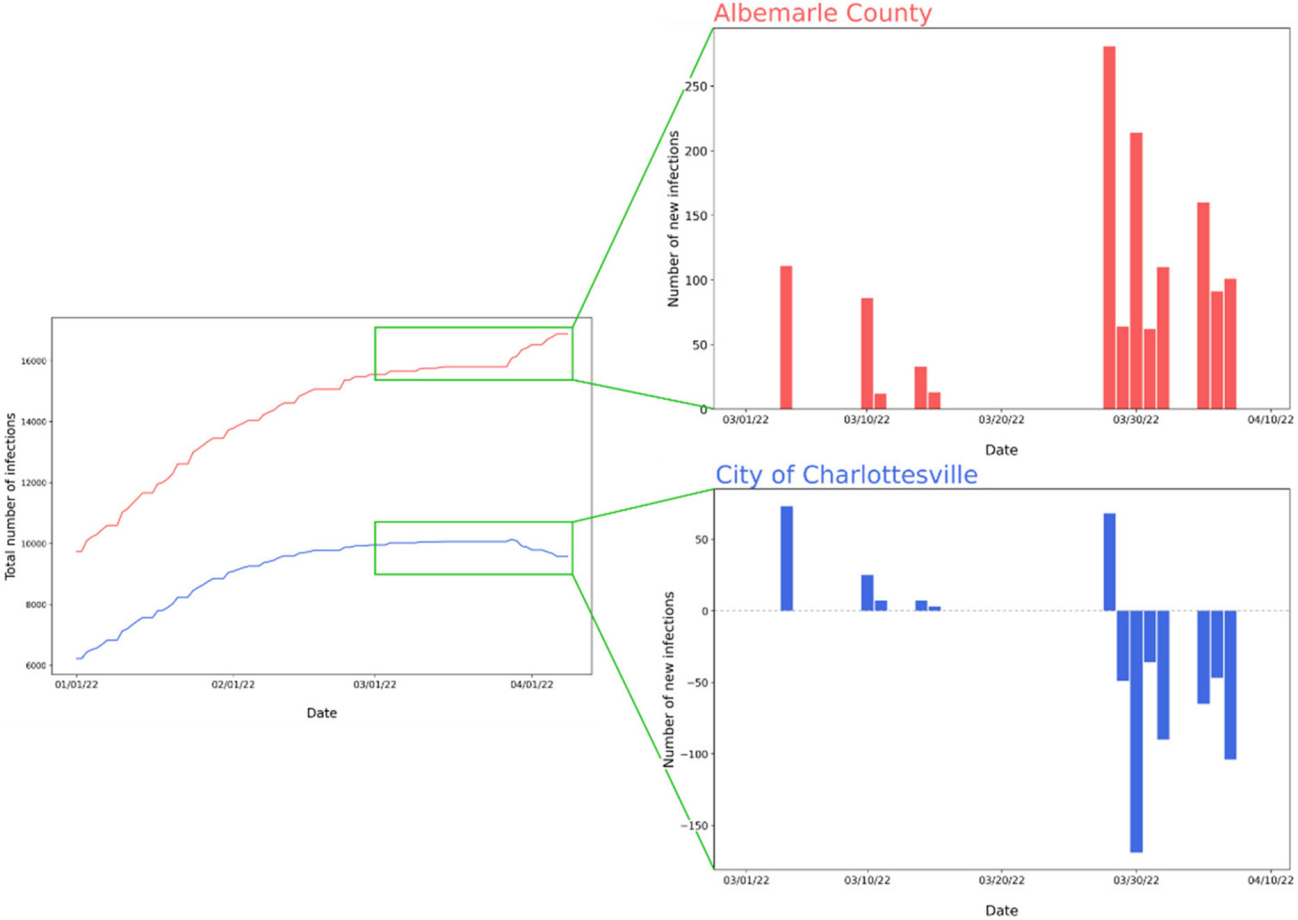
The total number of COVID-19 infections in the city of Charlottesville and Albemarle County between January 1, 2022 and April 8, 2022. Data were obtained from USAFacts in June 2022.

The Daily Progress^53^ identified another problem with the design of databases, which was illustrated using an example of the data from two localities in Virginia: the City of Charlottesville and Albemarle County. Between March and April 2022, the local health district of the Virginia Department of Health (VDH)^54^ attempted to correct past mistakes in the area's COVID-19 infection data by retroactively reassigning the correct locality to old cases. We used the data acquired from USAFacts to recreate the figure presented in the article (based on data from VDH). The general observation pointed out in *The Daily Progress* article holds true. The number of total COVID-19 infections from the beginning of the pandemic has been decreasing in Charlottesville, which cannot be correct (Fig. 10). The coronavirus cases from Charlottesville did not disappear, but they were gradually added to the count for Albemarle County. In the data from USAFacts, there was no information about when the infections occurred, and they add new cases with the date they received the data or updated their database. Additionally, USAFacts does not provide a mechanism to revise prior data, and instead updates have to be submitted as if they were “new” cases. Furthermore, we can notice differences in the plot from the article in The Daily Progress and the plot in our paper—the reason for the differences is the use of different data sources, VDH and USAFacts. Based on them, we can speculate that USAFacts is provided with information on new cases less frequently than VDH, which focuses on Virginia, so it may stay in closer contact with hospitals, clinics, and testing facilities in this area.

**FIG. 11.**
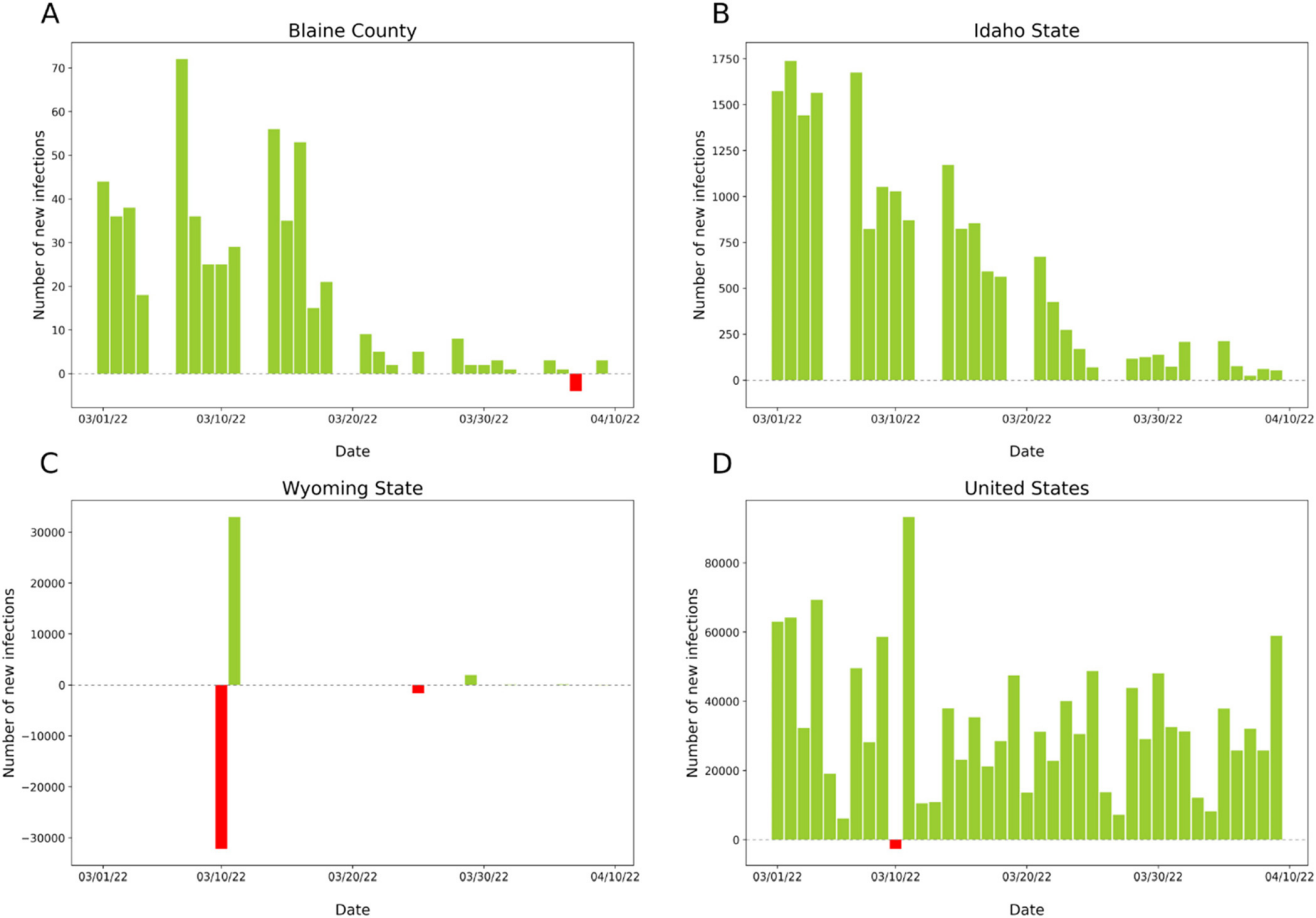
A number of daily COVID-19 infections in Blaine, Idaho, Wyoming States, and in the USA between March 1, 2022 and April 8, 2022. Data were obtained from USAFacts in June 2022.

The problem with infection count did affect not only the city of Charlottesville and Albemarle County but also other counties as well. A negative daily number of infections was observed in Blaine County in Idaho (Fig. 11). In this case, it cannot be determined to which locality the cases of infection were added or whether there was another reason for the situation. It is possible that an error occurred earlier and was corrected later by simply subtracting the surplus infections. Thus, we cannot be sure how many infections really happen each day. Looking at Figs. 10 and 11, there are also days when no new cases were reported, but we can assume that the database was not updated on those days. One should realize that the virus does not celebrate neither holidays nor weekends.

**FIG. 12.**
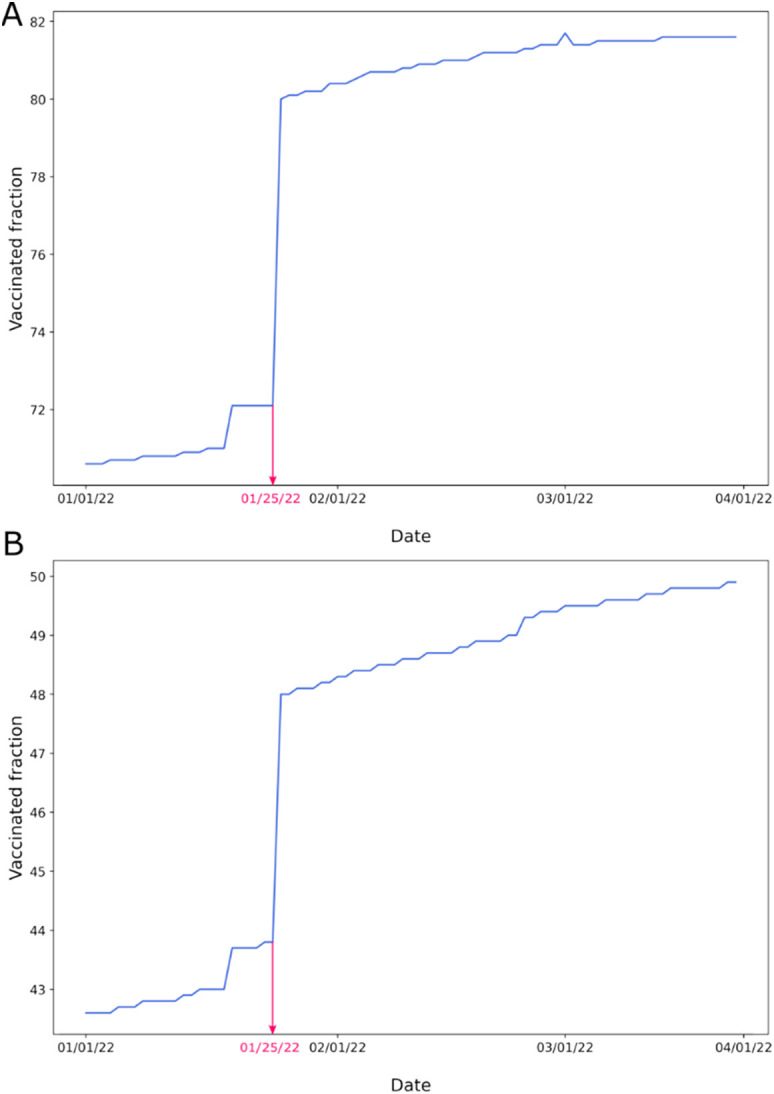
Percentage of fully vaccinated population in (a) Blaine County and (b) Twin Falls County in Idaho between January 1, 2022 and March 22, 2022. Data were obtained from CDC in June 2022.

Furthermore, we noted problems with the vaccination data. Blaine County and its neighbors had a sudden increase in the vaccination rate on January 25, 2022 (Fig. 12). Blaine County's vaccination rate increased by almost 8% and Twin Falls' by more than 4%. The sudden rise occurred because recipients below 18 years old were excluded from calculations before January 25 and were considered only after that date. Before January 25, the state of Idaho was not providing vaccine data to the CDC for people younger than 18 years old.^55^ This change across the state resulted in a single day “increase” of 177 996 doses administered. This example shows the problem with the design of the CDC database. Instead of recording the date when a person received their vaccination, the databases contained a date of entry or update of this particular record. This makes it difficult to know if the vaccination has just happened or has been effective for a while, thus making it very difficult to analyze vaccine efficacy as a function of time.

**FIG. 13.**
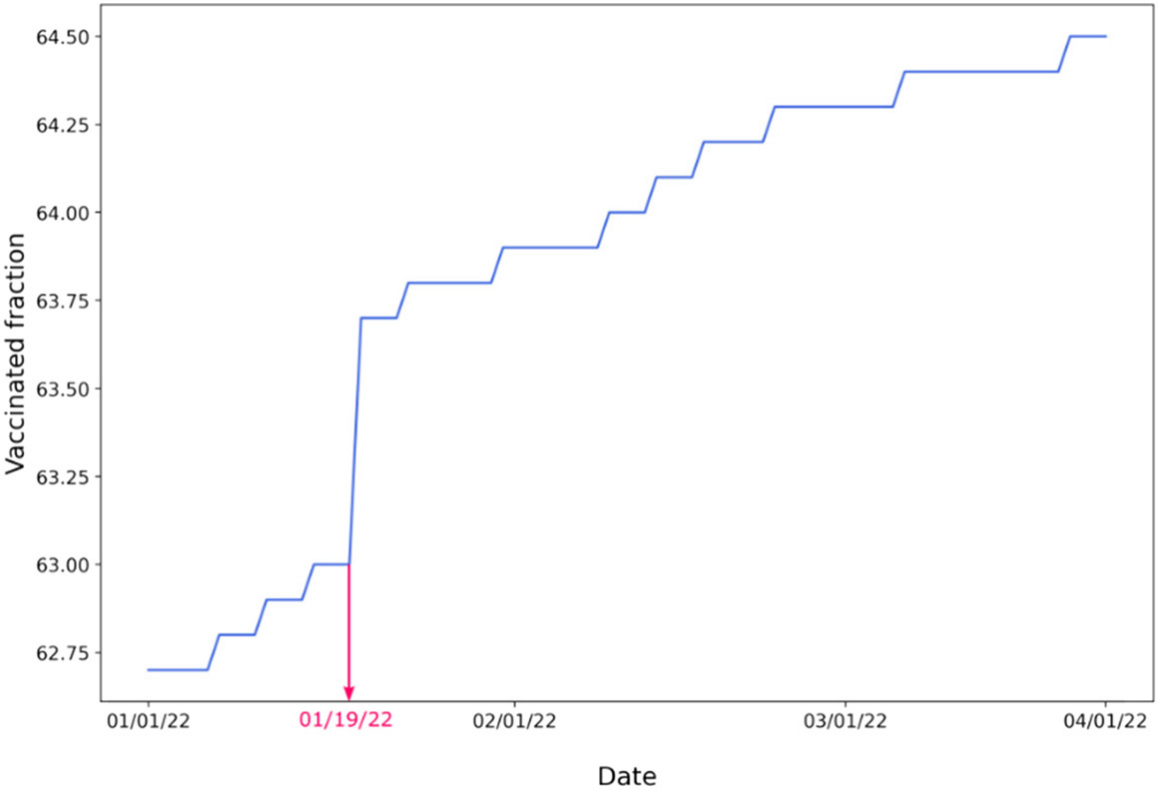
Percentage of fully vaccinated population in Albemarle County in Virginia between January 1, 2022 and April 9, 2022. Data were obtained from CDC in June 2022.

On January 19, 2022, the CDC changed the address algorithm for the Bureau of Prisons (BOP) and Department of Defense (DOD) to align vaccine administration counts with the residential county of the recipient instead of the county where the vaccine was administered.^56^ This change significantly impacted the county-level vaccination rate in different localities. That means that there was a sudden increase in the vaccination rate in some regions and a decrease in others. We encountered that problem while analyzing the vaccination rate in Albemarle County, Virginia (Fig. 13). On January 19, there was an almost 1% rise.

### Design of an Advanced Information System (AIS) to battle future pandemics

Several parties, including researchers, facilities, developers, funding agencies, and governing bodies, must cooperate to ensure that the above-mentioned problems are avoided in future pandemics. Effective transformation of information and data into knowledge is challenging and requires a new way of viewing resources and databases. In our previous work, we presented recommendations^41^ that should be coordinated on several levels involving different organizations. These suggestions could serve as a foundation for creating an Advanced Information System (AIS),^57,58^ ensuring a rapid and efficient response plan for future pandemics. The AIS will facilitate acquiring data from disparate sources (resources and databases) through a sophisticated system of connections and will have carefully designed databases that are more resistant to problems and exceptional cases. The AIS would also ensure that data are aggregated correctly. Scientists with expertise in different domains must collaborate to create the suggested strategies and policies.

## DISCUSSION AND CONCLUSIONS

The COVID-19 pandemic significantly impacts global health, economies, and societal norms. The rapid spread of the virus and the uncertainty surrounding its transmission, severity, and potential treatments have led to widespread fear and disruption. One problem with data collection and aggregation is that early forecasts of COVID-19 cases and mortality in some states and counties were found to be inaccurate due to inconsistencies or errors in how data were interpreted, reported, and combined. This highlights the importance of accurate and timely data collection and reporting in understanding the spread and impact of the virus.

We analyzed anomalies in the infection, mortality, and vaccination data collected by the main COVID-19 data sources. The abnormalities that have been found can result from how the data were aggregated, delays in data collection, or sudden changes in the system of how the numbers are counted. Ensuring the correctness of collected data is extremely important for data analysis. Garbage in, garbage out is a broadly known concept in data management that must be considered in every analytical work. Flawed data and the methodology by which datasets are prepared and built can produce nonsense results that may lead to serious consequences. The response of governments to a crisis may inadvertently be affected by misconceptions in conclusions drawn from poorly constructed datasets.

Providing raw data is one technique to aid data analysts in analyzing data generated very quickly and challenging to retain under a lot of human control. A data analyst concerned about a particular component of the processed data could refer to the source and find the reason behind observed abnormalities. Moreover, by examining raw data, finding reporting delays can be possible. These changes could improve the accuracy of data analysis. Additionally, in our previous paper,^41^ we recommended actions that can be taken by different parties to unify and revamp the response to a crisis. We suggested implementing advanced information systems that can be used to improve communication and coordination among healthcare providers and public health officials, streamlining the response to a pandemic.

There are two striking situations. In a major European country, a high school student found that the sum of the number of COVID-19 cases from all provinces did not agree with the total number of cases provided by the government agency responsible for aggregating the data. Instead of fixing the simple summation problem, the government made province-level data unavailable to the public, effectively sweeping the problem “under the rug.” In the same country, the number of excessive deaths was much higher than the official number of COVID-19 fatalities. As described earlier, in North America, the number of total cases in some places decreased over time.

Scientific papers that are cited thousands of times are considered a great success in the academic world. Still, in the real world, the misinformation stream pumped through social media and charismatic skeptics can instill fears much faster than scientific communications can assuage them. Clearly, we are able to mobilize rapidly; 300 000 papers on SARS-CoV-2 since the start of the pandemic demonstrate that. This is a great success in itself, but why are we not as good as we could be? We are making contributions instead of making a breakthrough.

## Data Availability

The data that support the findings of this study are available within the article.
